# Human SOD2 Modification by Dopamine Quinones Affects Enzymatic Activity by Promoting Its Aggregation: Possible Implications for Parkinson’s Disease

**DOI:** 10.1371/journal.pone.0038026

**Published:** 2012-06-18

**Authors:** Elisa Belluzzi, Marco Bisaglia, Elisabetta Lazzarini, Leandro C. Tabares, Mariano Beltramini, Luigi Bubacco

**Affiliations:** 1 Department of Biology, University of Padova, Padova, Italy; 2 CEA, Institut de biologie et de technologies de Saclay, Service de Bioénergétique, Biologie Structurale et Mécanismes, Gif-sur-Yvette, France; INSERM/CNRS, France

## Abstract

Mitochondrial dysfunction and oxidative stress are considered central in dopaminergic neurodegeneration in Parkinson’s disease (PD). Oxidative stress occurs when the endogenous antioxidant systems are overcome by the generation of reactive oxygen species (ROS). A plausible source of oxidative stress, which could account for the selective degeneration of dopaminergic neurons, is the redox chemistry of dopamine (DA) and leads to the formation of ROS and reactive dopamine-quinones (DAQs). Superoxide dismutase 2 (SOD2) is a mitochondrial enzyme that converts superoxide radicals to molecular oxygen and hydrogen peroxide, providing a first line of defense against ROS. We investigated the possible interplay between DA and SOD2 in the pathogenesis of PD using enzymatic essays, site-specific mutagenesis, and optical and high-field-cw-EPR spectroscopies. Using radioactive DA, we demonstrated that SOD2 is a target of DAQs. Exposure to micromolar DAQ concentrations induces a loss of up to 50% of SOD2 enzymatic activity in a dose-dependent manner, which is correlated to the concomitant formation of protein aggregates, while the coordination geometry of the active site appears unaffected by DAQ modifications. Our findings support a model in which DAQ-mediated SOD2 inactivation increases mitochondrial ROS production, suggesting a link between oxidative stress and mitochondrial dysfunction.

## Introduction

Parkinson disease (PD) is a multifactorial neurodegenerative condition characterized by the progressive loss of dopaminergic neurons in the *Substantia Nigra pars compacta* and by the presence of intracellular inclusions, known as Lewy bodies, in surviving neurons. The role of oxidative stress and mitochondrial dysfunction in the pathogenesis of PD has received widespread interest [1]. Oxidative stress occurs when the capacity of the endogenous antioxidant systems are overcome by the generation of reactive oxygen species (ROS), which ultimately leads to cellular damage and death. A possible mechanism involved in the increase of oxidative stress, which could account for the preferential degeneration of dopaminergic neurons in PD, involves the redox reactions specific of dopamine (DA). Several different pathways have been identified for the oxidation of DA [2]. These reactions lead to both toxic ROS and dopamine-quinones (DAQs) [3]. ROS can damage cellular components such as lipids, proteins, and DNA [4]. The electron-deficient quinones can also react with cellular nucleophiles, such us cysteine residues, leading to further cytotoxicity. DAQ modifications on tyrosine hydroxylase, the rate-limiting enzyme in DA synthesis, or parkin, whose coding sequence PARK2 is mutated in familiar PD, have been described *in vitro* and *in vivo* along with important inhibiting effects on protein functions [5], [6]. We recently characterized the structural modifications and functional effects induced by DAQs on DJ-1, a protein involved in PD proposed to act as an oxidative stress sensor [7], and on α-synuclein, a protein involved in familiar forms of PD and the major component of Lewy bodies [8], [9], [10].

In the present study, we were interested in verifying a possible involvement of mitochondrial superoxide dismutase 2 (SOD2) in the pathogenesis of PD. ROS are produced under physiological conditions, in particular in the mitochondrial oxidative phosphorylation process, at levels that allow scavenging by endogenous antioxidants such as superoxide dismutases, glutathione peroxidase, catalase, and small molecules such as vitamins C and E and glutathione. Among the ROS-scavenging enzymes, SODs are often regarded as the first line of defense [11]. These proteins convert naturally occurring superoxide radicals to molecular oxygen and hydrogen peroxide. Three different SOD isoenzymes that are well compartmentalized have been characterized in humans. SOD1 is located in the cytosol as well as in the mitochondrial intermembrane space, but it is also present in the peroxisomes and in the nucleus of cells [12]. SOD2 is a mitochondrial manganese enzyme, which acts as the main scavenger of superoxide anions within mitochondria [12]. SOD3 is an extracellular protein which is expressed in specific cell types and tissues, such as vascular smooth muscular cells, lung, and plasma [12].

Considering its mitochondrial localization, SOD2 appears to be the most relevant in the context of PD. Two different studies by the same group, based on proteomic analyses of isolated rat brain mitochondria following exposure to DAQs, suggested a direct interaction between SOD2 and the brain mitochondria. In the first one, by using a combination of fluorescent probes directed against cysteine or lysine residues, the investigators demonstrated that DA oxidation results in the loss of mitochondrial proteins, among which SOD2 [13]. In the second study, SOD2 was identified as one of the proteins modified by ^14^C-DAQs in rat brain mitochondria [14]. Nevertheless, the molecular mechanisms of this interaction remain largely unexplored, as well as the potential functional effects induced by such modifications.

In our study, we analyzed the interaction between SOD2 and DAQs *in vitro*. By analysis of the relevant point mutants, we identified the protein residues modified by the DA oxidation products. Specifically, Cys196 appears to be the SOD2 residue involved in the reaction with the quinone species. Exposure to DAQs induces loss of SOD2 enzymatic activity, which is correlated to the concomitant formation of protein aggregates. The *in vitro* data presented here support the hypothesis of a role for SOD2 in the progression of PD. As with all models, this proposal needs to be tested in relevant cellular and animal models.

## Results

### Interaction between SOD2 and DAQs

**Figure 1:**
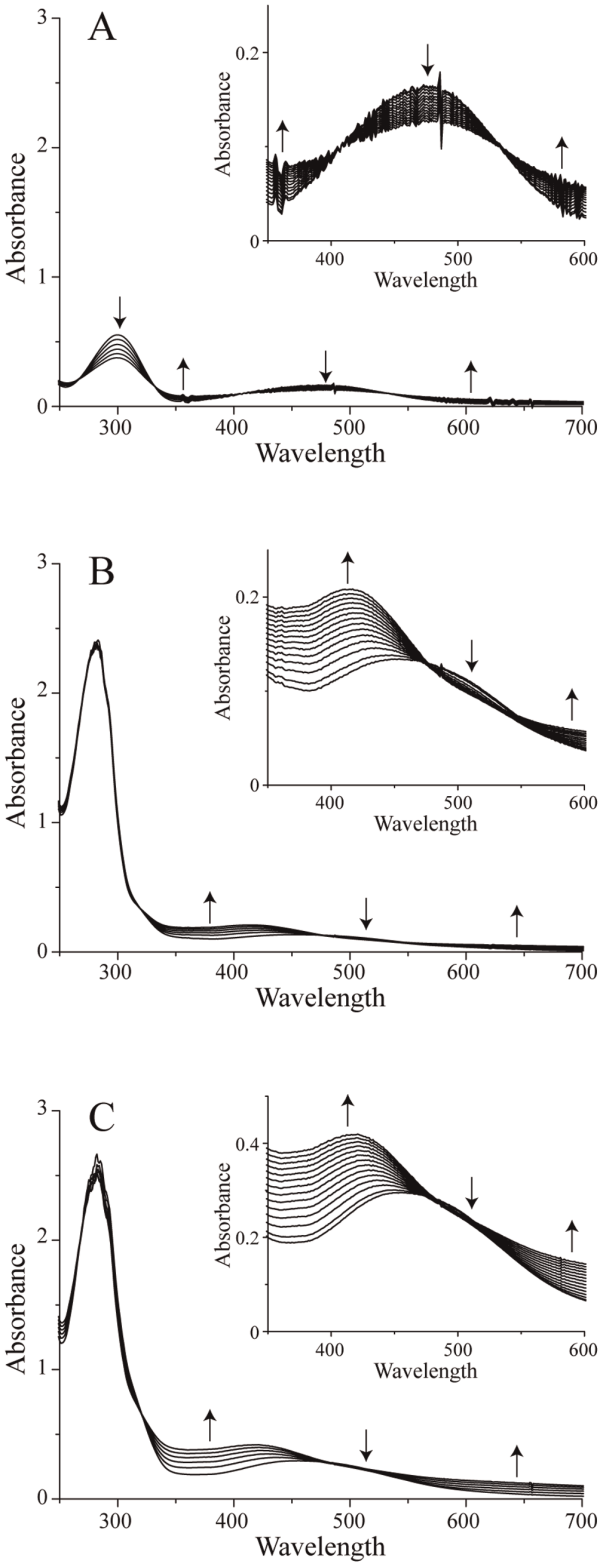
UV-Vis analysis of DAQs reactivity toward SOD2. To make the graphical visualization easier, only a few selected spectra are shown, recorded at 10 minute intervals after the addition of Ty in the full spectra and every 4 minutes in the insets. A) DA alone, B) DA and SOD2 in an equimolar ratio, and C) DA and SOD2 in a molar ratio of two. Upward arrows and downward arrows indicate, respectively, a time-dependent increase and decrease of the spectral features.

To assess whether DAQs could react with SOD2, the reactions were first followed by UV-vis spectroscopy. The use of tyrosinase (Ty) to rapidly produce DAQs offers the advantage of preventing the formation of radical species in solution. Ty uses molecular oxygen to catalyze two different enzymatic reactions, the hydroxylation of monophenols to diphenols (monophenolase activity) and the oxidation of diphenols to quinones (diphenolase activity). Both reactions take place without the production of radicals using molecular oxygen as cosubstrate [15]. The oxidation of DA generates three monomeric quinone species, dopamine-o-quinone (DQ), aminochrome (AC), and indole-5,6-quinone (IQ) [10], which are all potentially reactive in a cellular environment. While the spectroscopic characterization of IQ is still elusive, DA, DQ, and AC possess characteristic UV-Vis peaks with absorption maxima at 280 (DA), 390 (DQ) and 300/480 (AC) nm [3]. As previously reported [3], during the Ty-mediated oxidation of DA at physiological pH and 25°C, only AC is detectable in the optical spectra with no evidence of the presence of DQ. This can be explained by the basis of the high reactivity of DQ that undergoes an intramolecular cyclization reaction with a rate constant of ∼0.15 s^−1^ [16]. On the contrary, the rearrangement reaction which involves AC to form 5,6-dihydroxyindole (DHI), with a rate constant of ∼10^−3^ s^−1^, is much slower, leading to accumulation of AC [10]. In Fig. 1A, the spectra recorded after the addition of Ty to a solution containing DA are reported; the peaks centered at 300 and 480 nm reveal the formation of AC. As already described [10], [17], light scattering increases during the reaction as a consequence of the polymerization process.

It has been proved that the rate constant of the nucleophilic addition of free cysteine or glutathione to DQ is three orders of magnitude faster than that of the cyclization reaction, with a value higher than 200 s^−1^ [16]. It follows that SOD2, with its two free cysteine residues, is a suitable target of DAQs. After verifying that Ty does not react directly with SOD2 by UV-Vis spectroscopy (data not shown), we analyzed the reactivity of DAQs towards the protein. Following the addition of a catalytic amount of Ty to a solution containing SOD2 and DA in a 1∶1 molar ratio, *i.e*., a substoichiometric ratio of DA to cysteine residues, the appearance of the peak corresponding to AC, centered at 480 nm, revealed that the newly formed DQ does not react with the protein (Fig. 1B). A comparison of the SOD2:DA mixture experiment with the control, reported in Fig. 1A, revealed the formation of a new peak around 420 nm that appears after AC formation. A very similar result was obtained working with a SOD2:DA = 1∶2 molar ratio (Fig. 1C). The only difference is shown by the intensity of the new peak, which is doubled (inset). In conclusion, the data obtained with SOD2 suggest that, while DQ does not react with the protein, some DA-derived species, subsequent to AC formation, do. This analysis does not allow us to distinguish whether only one or both cysteine residues are involved in the interaction with DAQs. Moreover, as the observed spectral feature at 420 nm has not been assigned to a specific molecule, also the nature of the modification induced on SOD2 after the DAQs formation remains unclear.

**Figure 2:**
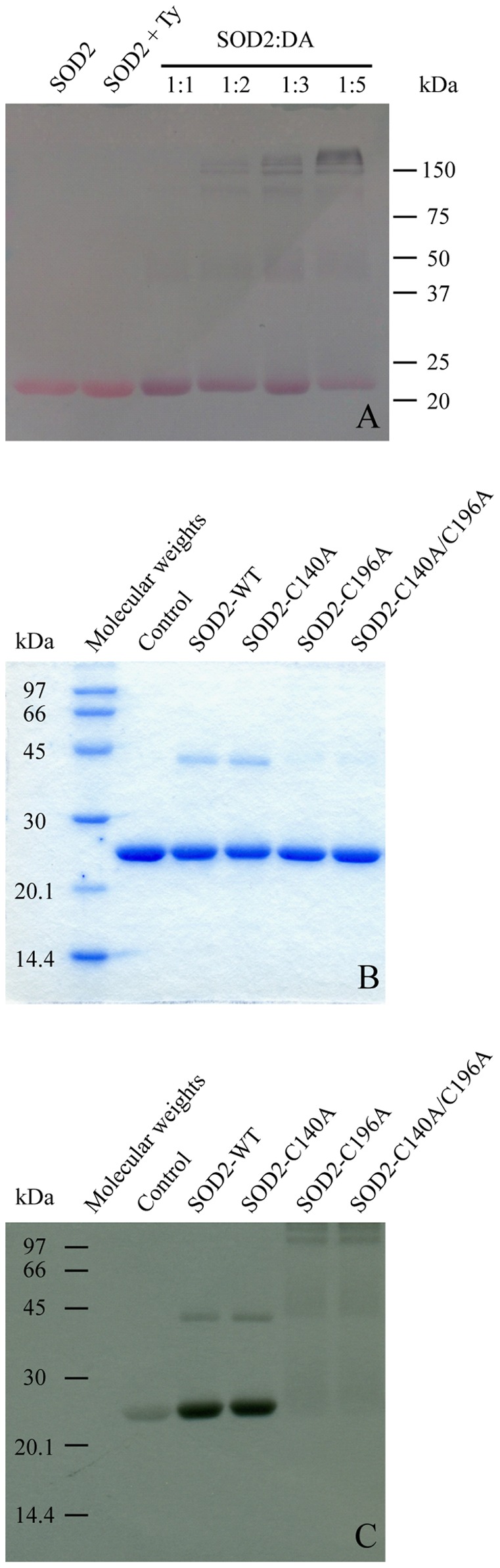
DAQs covalently modify SOD2. A) The redox-cycling staining of SOD2 before and after the reaction with DAQs at different molar ratios. The pink-red ponceau coloured SOD2-corresponding band becomes purple in the presence of DAQs. B) SDS-PAGE of SOD2 and its C-to-A mutants after the reaction with an equimolar amount of ^14^C-DA in the presence of tyrosinase. In the control, the reaction between SOD2 and DA was carried out without tyrosinase. C) The autoradiogram of the SDS-PAGE. The spots indicate the covalent incorporation of radioactivity into the protein.

For this reason, to independently verify the presence of DAQs/SOD2 adducts, the protein was incubated with DA in the presence of Ty and the reaction products were successively analyzed by SDS polyacrylamide gel under denaturing conditions. The gel was developed using the redox-cycling staining technique, which exploits the ability of quinones and related quinoid substances to catalyze redox cycling at an alkaline pH in the presence of excess glycine as a reductant [18]. The released superoxide reduces nitroblue tetrazolium to the coloured dye formazan (blue-purple colour), which allows the detection of proteins containing quino-compounds [18]. The reactivity of SOD2 towards the oxidation products of DA was confirmed by showing the formation of SOD2 monomeric species covalently bound to quinoid compounds (Fig. 2A). Increasing the DA:protein molar ratio, new bands corresponding to high molecular weight species are also visible.

To further confirm the formation of covalent bonds between SOD2 and DAQs and to evaluate the role of each cysteine residue, a radioactivity assay was performed on wild-type (wt) SOD2, as well as on Cys-to-Ala mutant proteins, to easily detect the formation of quino-complexes and make sequence assignments. Proteins were exposed to ^14^C-DA in the presence of tyrosinase, in a 1∶1 protein to DA ratio. The reaction products were first separated by SDS-PAGE (Fig. 2B) and then detected by autoradiography (Fig. 2C). Distinct spots of radioactivity indicated protein targets covalently modified by ^14^C-DAQs. The autoradiogram was then aligned with the corresponding SDS-PAGE gel for DAQ-modified protein identification. Incorporation of radioactivity into the spot corresponding to the wt SOD2 indicated the formation of SOD2/DAQ covalent adducts following the generation of quinone species. Moreover, a faint band corresponding to the formation of covalent dimeric DAQs-modified species is also visible in addition to the spot relative to the monomer. When SOD2 and DA were incubated in the absence of Ty, radioactivity was still incorporated into the monomer, although to a lower extent. This observation suggests that, while Ty is important to allow the rapid generation of quinone species, the oxidation chemistry of DA to form quinones is possible in a cellular context where cytosolic Ty is most likely absent or very low [19].

The C140A mutant showed a pattern of spots analogous to the wt protein, while the results obtained with the C196A and C140A/C196A double mutant were very different. With these two mutants, we did not observe radioactive spots corresponding to monomers or dimers, while faint radioactive bands appeared at high molecular weights. A possible explanation is that a small amount of protein was trapped in the neuromelanin-like aggregates, formed during the polymerization process, which involves DAQs.

### Enzymatic Activity Modulation by DAQs

Starting from the observation of a covalent bond between DAQs and one cysteine residue of SOD2, we analyzed whether the enzymatic activity of the protein was affected by this interaction. To this aim, we performed an indirect assay based on the auto-oxidation of pyrogallol, which occurs at alkaline pH and produces superoxide anions. This method has been reported in the literature as one of the best in terms of reproducibility, accuracy, and simplicity [20]. The mechanism of the auto-oxidation involves the superoxide anion as a propagating species, which leads to the formation of purpurogallin, which can be followed at 420 nm. When SOD2 is present, it reacts with and removes the superoxide anions, inhibiting the auto-oxidation of pyrogallol and the purpurogallin formation. Pyrogallol auto-oxidation occurred also in the aqueous stock solution; to overcome this problem, the stock solution was prepared by dissolving pyrogallol under anaerobic conditions.

**Figure 3:**
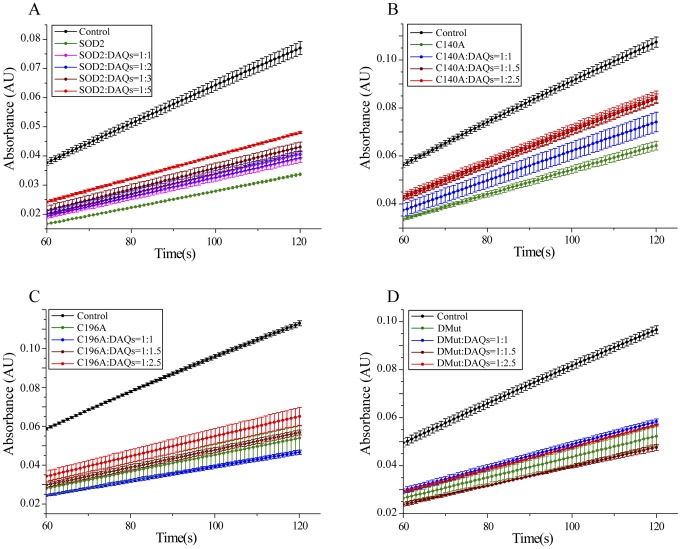
Effects of DAQ-induced modification on SOD2 activity. Pyrogallol-assay performed on A) wt SOD2, B) C140A mutant, C) C196A mutant, and D) C140A/C196A mutant.

**Figure 4:**
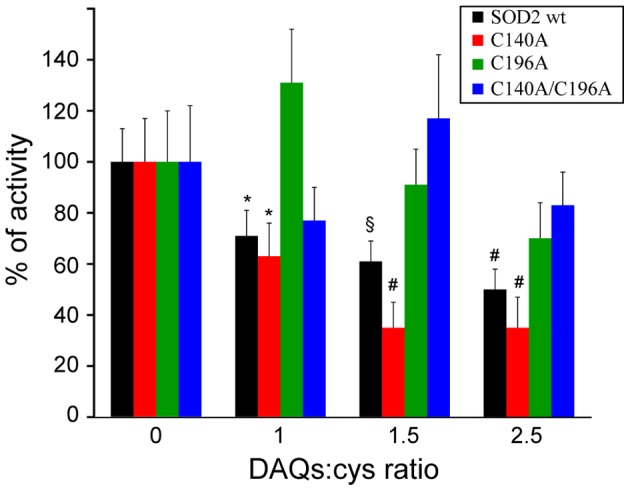
DAQ-induced modification on C196 affects SOD2 activity. The comparison of the residual activity after DAQ-modification indicates that the activity of wt SOD2 and the C140A mutant decreases significantly in the presence of DAQs in a dose-dependent manner. The activity of C196A and C140A/C196A mutants does not show a statistically significant DAQ-dependent effect. Values are the mean ± SD. * p<0.05, § p<0.01, # p<0.001 relative to the protein activities before the reaction with DAQs.

Fig. 3A shows the kinetic profiles recorded for the auto-oxidation of pyrogallol as control and in the presence of SOD2, before and after the reaction with DAQs at different molar ratios. As expected, in the presence of SOD2, the kinetics of purpurogallin formation was inhibited, as indicated by a decrease of the absorbance observed. DAQ-induced SOD2 modifications caused an increase of pyrogallol auto-oxidation with respect to the unmodified protein, demonstrating that DAQ reaction with SOD2 results in an inhibition of its enzymatic activity. The extent of the inhibition depends on amount of DA used in the reaction and therefore on the fraction of modified SOD2 molecules. The decrease of the SOD2 activity ranges from 25±6% in the presence of an equimolar amount of DAQs to 50±3% with a fivefold excess of DAQs, as summarized in table 1. To evaluate if the DAQ-modification of C196 was sufficient to reproduce the effects observed with the wt protein, we repeated the enzymatic assays on the single and double Cys-to-Ala SOD2 mutants. The DAQ concentrations used to treat the single mutants were adjusted to keep the cysteine to DA ratio for the single mutants comparable to the wt protein (Fig. 3B–D). The comparison of the residual enzymatic activities after DAQ-modification of wt, C140A, C196A and the double mutant C140A/C196A are reported in Fig. 4. The statistically significant DAQ-dependent inhibition of the enzymatic activity observed for the wt protein was clearly reproduced only with the C140A mutant. On the contrary, the activities of C196A and C140A/C196A mutants do not show a statistically significant DAQ-dependent effect.

**Table 1:** The kinetic parameters measured in the pyrogallol assay and the calculated activities for SOD2, before and after the reaction with DAQs at different molar ratios.

	Slope (AU/s) •10^4^	% of inhibition	Activity	Specific activity (U/mg) •10^2^	% activity
Control	6.6±0.2	–	–	–	
SOD2	2.83±0.06	57±4	1.33±0.12	4.4±0.4	100±13
SOD2:DAQs = 1∶1	3.31±0.11	50±14	0.99±0.11	3.3±0.3	75±10
SOD2:DAQs = 1∶2	3.40±0.07	48±4	0.94±0.10	3.1±0.3	71±10 *
SOD2:DAQs = 1∶3	3.63±0.03	45±3	0.82±0.09	2.7±0.3	61±8 ^§^
SOD2:DAQs = 1∶5	3.96±0.07	40±3	0.67±0.08	2.2±0.3	50±8 ^#^

* p<0.05, ^§^ p<0.01, ^#^ p<0.001 relative to the SOD2 activity before the reaction with DAQs.

### Effects of DAQs on the Mn(II) Active Site and on Aggregation

As a final objective of the present study, we tried to correlate the loss of functional activity with structural modifications induced on the protein by DAQs. At least two possible models could explain the loss of activity observed in our functional experiments. First, DAQs could induce structural changes on the active site affecting enzymatic activity. The second model relies on more drastic structural effects induced by DAQs that promote precipitation or the formation of high molecular weight aggregates.

**Figure 5:**
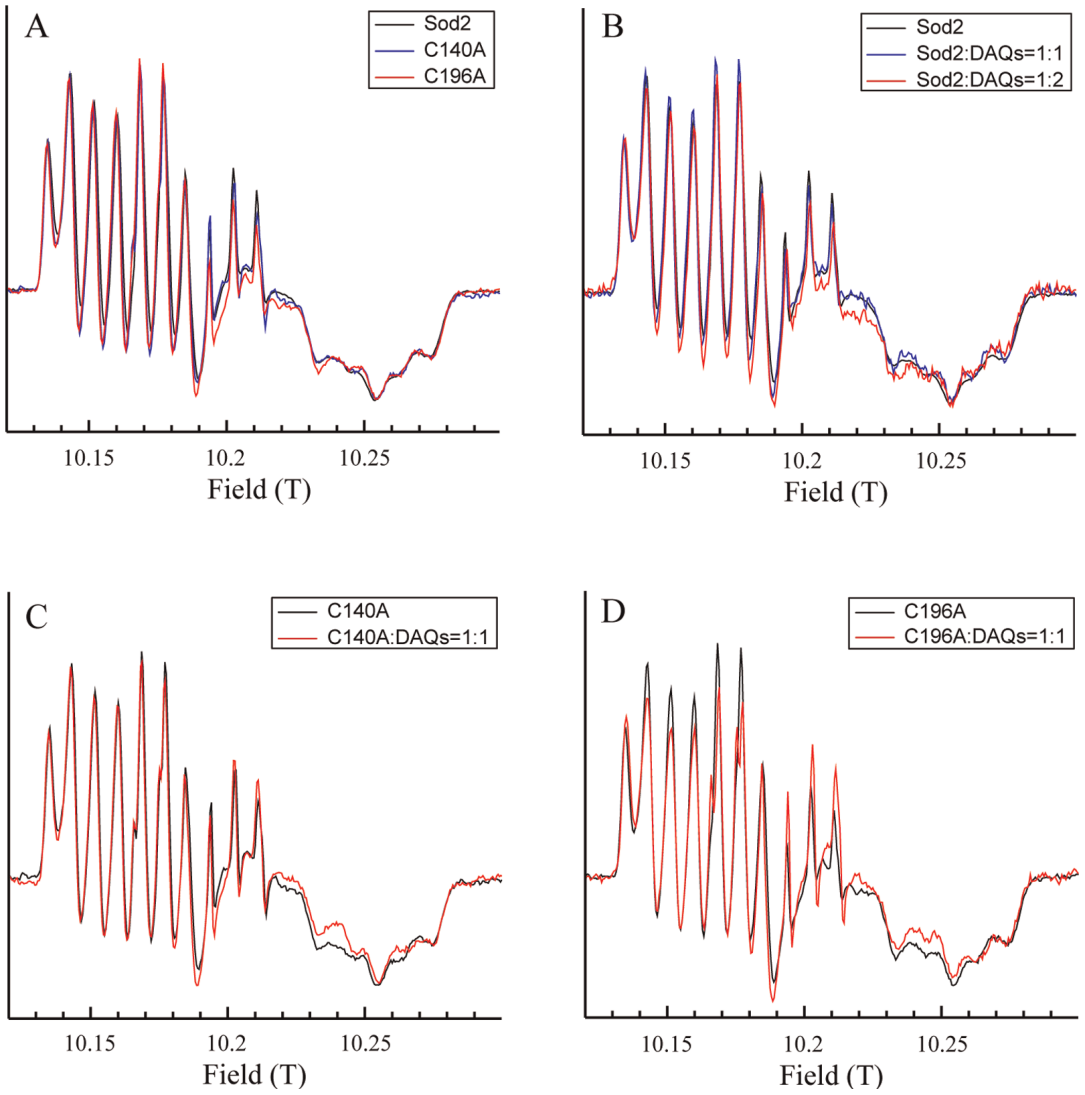
DAQ-induced modification does not affect the Mn(II) binding site of SOD2. 285 GHz HFEPR spectra of A) wt SOD2, B) C140A mutant, C) C196A mutant, and D) C140A/C196A mutant, at 25 K before and after the reaction with DAQs. Spectra have been arbitrarily scaled for better comparison.

To verify whether DAQs could affect the enzymatic properties of SOD2 by modifying the active site of the protein, high-field high-frequency electron paramagnetic resonance (HFEPR) experiments were recorded on wt SOD2 and on its C140A and C196A single mutants. It has been shown that HFEPR produce highly resolved Mn(II) spectra from which it is possible to determine all the spin parameters with high accuracy. HFEPR is so sensitive to the Mn(II) electronic structure that using this technique, it is possible to observe differences among distinct SODs even if their crystallographic structures are virtually identical [21], [22], [23]. For this reason, HFEPR is the technique of choice to investigate whether the Mn(II) site of SOD2 is modified after reaction with DAQs. We first analyzed the effects of the Cys-to-Ala mutations on the metal binding site of the protein. As shown in Fig. 5A, the HFEPR spectra of the C146A and C196A mutants are superimposable on that of wt protein, indicating that these mutations do not affect the structure of the active site. Figure 5B shows the HFEPR spectrum of wt SOD2 together with those of the protein after reaction with DAQs at 1∶1 and 1∶2 ratios. The human wt SOD2 HFEPR spectrum shows the same overall shape of previously published Mn(II) HFEPR spectra of other SODs and closely resembles that of the Mn-SOD from *E. coli*, falling into the category of the Mn-Type HFEPR spectrum [15]. After reaction with DAQs, the HFEPR spectrum shows no relevant changes, indicating that in the DAQ-modified protein, the Mn(II) sites have the same structure as in the wt protein. Moreover, the resemblance is such that structural changes at the substrate access channel may be ruled out since it has been shown that mutations at this position, as well as the interaction with analogues of the substrate, have clear effects on the HFEPR spectrum [24], [25]. In addition, no evidence of spurious Mn(II) was observed in the HFEPR spectra. Attempts of reducing any possible oxidized manganese by adding 1 mM sodium dithionite only increased the intensity of the spectra with no other observable changes (data not shown). Similar conclusions can be extended to the analysis of the HFEPR spectra of the mutants recorded before and after the reaction with DAQs at 1∶1 ratio (Fig. 5C–D). Some small differences, which are visible for example in the splitting of the central lines of C196A:DAQ-1∶1, are a consequence of a small amount of spurious aqueous Mn(II) (less than 1%) which gives rise to a narrow six-line component underneath the broad component of SOD2 [24]. Despite this spurious Mn(II), the resonances positions of SOD2 were not affected by the DAQ modification indicating that the active site structure was conserved.

**Figure 6:**
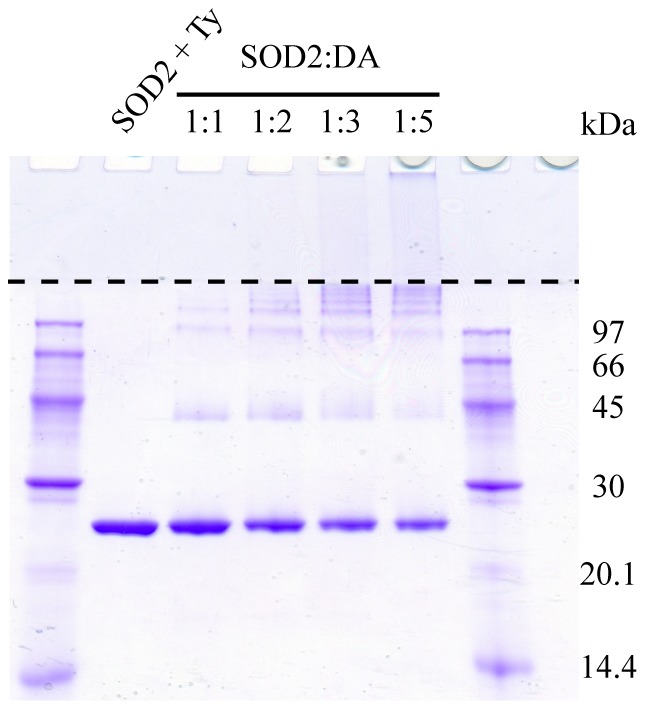
DAQ-induced modification affects SOD2 activity by inducing the formation of aggregates. SDS-PAGE of SOD2 before and after the reaction with DAQs at different molar ratios. The dashed line represents the separation between stacking and resolving layers of the polyacrylamide gel.

To evaluate the second model, the same samples used in the enzymatic assays were loaded and analyzed by SDS-PAGE under denaturing conditions (Fig. 6). Following the exposure of SOD2 to increasing amounts of DAQs, the intensity of the bands corresponding to the monomeric protein decreased while new bands appeared in the gel, as already observed with the redox-cycling staining (Fig. 2A). Densitometric quantification indicated that the intensity loss is approximately 15±6% in the presence of an equimolar amount of DAQs to 48±5% with a fivefold excess of DAQs. Bands corresponding to SOD2 dimers appeared in the presence of 1∶1 and 1∶2 SOD2:DAQ molar ratios, whose intensity decreased at higher DAQ concentrations, while the formation of high molecular weights aggregates was directly correlated to the DAQ concentration. These results strongly suggest that the apparent inhibition of SOD2 enzymatic activity could instead be correlated to the formation of protein aggregates.

## Discussion

The involvement of mitochondrial dysfunction and oxidative stress in the pathogenesis of Parkinson’s disease is now well accepted, as well as the role of DA and its oxidation products in promoting oxidative stress inside cells [11], [26], [27], [28], [29]. Nevertheless, while DA itself could account for the preferential dopaminergic neuron degeneration observed in PD, it is unlikely that it is responsible for the elevated oxidative conditions found in PD patients. Even under pathological conditions, DA concentration is expected to be low because of its MAO-dependent degradation, the active transport into vesicles by VMAT2, and its trapping inside neuromelanin [28]. One possibility is that chemical modifications induced by DA oxidation products on very specific protein targets could affect their function and result in an amplification of the effects of DA itself. Recently, by means of two different experimental approaches, it has been shown that SOD2 is a target of DAQs in rat brain mitochondria [13], [14], supporting the idea that a DAQ-induced decrease of the enzymatic activity of SOD2 would exacerbate oxidative stress leading to neuronal dysfunctions and eventually to cell death. In light of these results, the main objective of the present work was to establish a possible interplay between DA and SOD2 in the pathogenesis of PD.

The *in vitro* analysis of the interaction between SOD2 and the oxidation products of DA allowed the identification of the protein residues involved in the interaction with DAQs, emphasizing in particular the role of cysteine 196. The functional effect of DAQ modification is a loss of SOD2 enzymatic activity. Nevertheless, the effects were not drastic considering that even in the presence of a fivefold excess of DAQs, SOD2 retained approximately 50% of unmodified protein activity. Although it is hard to speculate about the SOD2 to DAQ molar ratio in a neuronal environment and in particular under pathological conditions, these data are in agreement with the progressive nature of the disease, which usually starts at an old age and slowly progresses over several years. Furthermore, SOD2 is required for normal biological function of tissues, and mice lacking SOD2 die within the first 10 days of life [30], so that the complete inactivation of the protein would have drastic effects.

The following step was to identify the structural basis of SOD2 inactivation induced by DAQ binding. Our results clearly indicate that DAQs promote the aggregation of SOD2 but have no effect on the coordination geometry of the Mn(II) binding site and do not promote the release of Mn(II). The HFEPR results may have two different interpretations: 1) in the DAQ-modified SOD2, the active site is unaffected and the enzymatic inactivation occurs via a different mechanism, or 2) the reaction with DAQs induce the oxidation of manganese to Mn(III) (or higher) which cannot be detected by HFEPR.

The neurotoxic effects of the oxidation products of DA and their ability to bind to sulfhydryl groups on protein cysteinyl residues were shown a long time ago by the pioneering studies carried out in the Hastings’ group [31], [32], [33] and, since then, several DAQ-modified proteins have been reported in the literature. For example, two different *in vitro* studies first demonstrated that DAQs covalently modify sulfhydryl groups of tyrosine hydroxylase, the rate-limiting enzyme in catecholamine biosynthesis, resulting in the formation of cysteinyl-catechols within the enzyme and loss of its enzymatic activity [5], [34]. More recently, DAQ binding to parkin, a ubiquitine ligase involved in autosomal recessive juvenile forms of PD [35], has been reported in dopaminergic cells [6]. The interaction between DAQs and the protein, which has been associated with a loss of ubiquitin ligase activity, has been demonstrated to promote parkin aggregation. Also in the case of α-synuclein, a protein associated to dominantly inherited forms of PD [36], we and others have demonstrated that DAQ interaction with the protein promotes the accumulation of high molecular weight SDS-resistant oligomers [9], [37]. In addition, DAQ-modified α-synuclein has been demonstrated to be poorly degradable by chaperone-mediated autophagy (CMA), and to block degradation of other substrates [38]. Very recently, we characterized the structural modifications induced by DAQs on DJ-1, a protein involved in PD, proposed to act as an oxidative stress sensor [7]. From all of these studies and in agreement with the data presented here, it appears that DAQ modifications may affect cell function in several ways, spanning from direct targeting of enzymatic or redox-sensing activities to protein degradation or protein aggregation.

One hypothesis that arises from this work is that DAQ-induced modification of SOD2 and the resulting decrease of enzymatic activity can amplify DA toxicity by promoting superoxide radical accumulation and, as a consequence, neuronal damage and death. The correlation that we propose between DAQs and SOD2 in the pathogenesis of PD could contribute to rationalize both the selective damage induced by DA to a dopaminergic neuronal population and the increased oxidative stress conditions observed.

As already emphasized, the objective of the present work was to unravel the molecular mechanism of the interaction between DA-derived quinones and SOD2, as well as the potential functional effects induced by such modifications. For this reason, the detailed *in vitro* analysis achieved on the isolated determinants seems very appropriate to the goal. Nevertheless, a more complex analysis in PD-relevant cellular and animal models is required to extend the proposed chemistry to cells.

In conclusion, if the route of inactivation of SOD2 unraveled here, together with the previously reported adduct formation in rat brain mitochondria, will be verified also in animal models, they may open new therapeutic perspectives.

## Materials and Methods

### SOD2 Preparation and Purification

Human wild-type SOD2 cDNA was amplified by PCR using the pCMV-SPORT6 vector (imaGenes), containing the full-length SOD2 coding region as template, and synthetic oligonucleotides (Sigma-Genosys), containing the *Nde*I and *Xho*I restriction sites. After digestion with the appropriate restriction enzymes, the PCR product was subcloned into the *Nde*I-*Xho*I linearized pET28a expression plasmid (Novagen), which contains a poly-histidine region followed by a thrombin cleavage site, and introduced into the *Escherichia coli* BL21(DE3) strain. The C140A, C196A, and C140A/C196A single and double mutants were generated by site-directed mutagenesis using specific oligonucleotides. Overexpression of the proteins was achieved in the *E. coli* BL21(DE3) strain by growing cells in LB medium at 37°C to an OD_600_ of 0.6 followed by overnight induction with 0.1 mM isopropyl β-D-thiogalactopyranoside in the presence of 3.65 mM MnCl_2_. After sonication and centrifugation, the soluble fraction containing SOD2 was purified using nickel affinity chromatography on a 1 ml HiTrap IMAC FF column (GE Healthcare). The purified proteins were digested with thrombin protease (Amersham Pharmacia) using the manufacturer’s protocol. After cleavage, the His-tag was separated from the protein using a cobalt resin His-Select® Cobalt affinity gel (Sigma-Aldrich). The final protein contained an extra G-S-H-M sequence at the N-terminus.

### UV-Vis Spectroscopy

Spectra were recorded on a personal computer-interfaced diode array Agilent 8453 UV-Vis spectrophotometer. Optical measurements were performed at 25°C using 100 µl HELLMA quartz cells with Suprasil windows and an optical path length of 1 cm. The wavelength range was 190–1100 nm. In a control experiment, 50 µM DA was dissolved in 50 mM Tris-HCl, pH 7.8, and spectra were recorded for 60 min at 30 s intervals after the addition of 3 units of mushroom tyrosinase (Ty) (Sigma-Aldrich). Similar experiments were then performed in the presence of 50 µM SOD2 with a 1∶1 or 1∶2 protein:DA molar ratio.

### NBT/Glycinate Redox-cycling Staining

DAQ-modified SOD2 was detected by redox-cycling staining [18]. Briefly, the protein samples, separated by SDS-PAGE, were transferred to nitrocellulose membranes at 50 V for 90 min at 4°C. The membrane was first stained with Ponceau S (0.1% in 5% acetic acid) resulting in a pink-red stain of each protein present. After washing with water, protein-bound quinonoids were detected by immersing the membrane in a solution of 0.24 mM NBT in 2 M potassium glycinate (pH 10.0) for 45 min in the dark resulting in a blue-purple stain of quinoprotein bands and no staining of other proteins. To block the staining, the membrane was immersed in 0.1 M borate buffer, pH 10.0.

### Radioactivity Assays

The reactions were performed in a final volume of 20 µl, at 25°C, in 50 mM Tris-HCl, pH 7.8, in the presence of 100 µM protein and 100 µM DA-8-^14^C (Sigma-Aldrich), containing 0.05 µCi of radioactivity. After the addition of 2 units of Ty, the reaction was carried out for 60 min. The reaction products (2 µl) were separated by 13% SDS-PAGE. After soaking in NAMP100 Amplify Fluorographic Reagent (GE Healthcare Life Science), gels were dried and detected by autoradiography.

### Indirect Activity Assay

Pyrogallol solution was prepared in dark conditions because the molecule is photosensitive. A volume of 8 ml of water and 30 mg of pyrogallol were mixed anaerobically to prevent pyrogallol auto-oxidation. The exact concentration of pyrogallol was then checked by measuring the absorbance at 267 nm using a molar extinction coefficient 1290.8 M^−1^ cm^−1^, previously determined in our laboratory. The assay was performed in 3 ml of 50 mM Tris-HCl, pH 8.2, in the presence of 1 mM EDTA and 1 µM catalase. When required, SOD2 was added to the solution to a final concentration of 45 nM. The reactions with DAQ were performed in 100 µl of 50 mM Tris-HCl, pH 7.8, using 2.5 units of Ty, in the presence of 40 µM of SOD2 and different protein:DA molar ratios. After the addition of pyrogallol to a final concentration of 0.2 mM, the auto-oxidation was followed for 120 seconds by measuring the absorbance at 420 nm with a diode array Agilent 8453 UV-visible spectrophotometer. Each measurement was repeated three times and data were elaborated between 60 and 120 seconds, which represents the range where the reaction is linear. Percent inhibition was calculated as follows:

% inhibition  =  [(control rate-sample rate)/control rate] x 100.

An arbitrary unit is usually defined as the amount of enzyme required to inhibit the auto-oxidation of pyrogallol by 50%. Data were analyzed using GraphPad Prism 4 software. One-way ANOVA followed by Tukey’s *post hoc* test was used to determine whether groups were statistically different. *P* values <0.05 were considered significant.

### EPR Spectroscopy

The high frequency electron paramagnetic resonance (HFEPR) spectrometer has been described in detail elsewhere [39]. Field calibration was based on a Mn(II)-doped MgO standard sample (g = 2.000101) [40], and the absolute error in field measurements was 1 G (0.1 mT) or 0.0001 in g. All spectra were obtained with 10 G modulation under non-saturating conditions at 285 GHz with 5 G resolution at 25 K. The quality of spectra was such that they could be reliably reproduced and singly and doubly integrated.

### SDS-Page Quantification

The same samples used in the enzymatic assays were loaded and separated by 13% SDS-PAGE under denaturing conditions. The dried gels were analyzed with ImageJ (Rasband, W.S., ImageJ, U.S. National Institutes of Health, Bethesda, Maryland, USA, http://rsb.info.nih.gov/ij/) for densitometry quantification. Experiments were performed in triplicate, and results are reported in the text as mean and standard deviation.
